# Cardio-Respiratory Endurance Responses Following a Simulated 3 × 3 Minutes Amateur Boxing Contest in Elite Level Boxers

**DOI:** 10.3390/sports6040119

**Published:** 2018-10-15

**Authors:** Said El-Ashker, Helmi Chaabene, Yassine Negra, Olaf Prieske, Urs Granacher

**Affiliations:** 1 Faculty of Physical Education, Mansoura University, Mansoura 35516, Egypt; sgelashker@iau.edu.sa; 2 Deanship of Preparatory Year, Imam Abdulrahman Bin Faisal University, Dammam 31441, Saudi Arabia; 3 Division of Training and Movement Sciences, Research Focus Cognition Sciences, University of Potsdam, Am Neuen Palais 10, 14469 Potsdam, Germany; chaabanehelmi@hotmail.fr (H.C.); prieske@uni-potsdam.de (O.P.); 4 Research Unit (UR17JS01), Sport Performance, Health & Society Higher Institute of Sports and Physical Education of Ksar Said, University of La Manouba, Manouba 2010, Tunisia; yassinenegra@hotmail.fr

**Keywords:** aerobic metabolism, physiological strain, striking combat sports, elite athletes

## Abstract

This study aimed at examining physiological responses (i.e., oxygen uptake [VO_2_] and heart rate [HR]) to a semi-contact 3 × 3-min format, amateur boxing combat simulation in elite level male boxers. Eleven boxers aged 21.4 ± 2.1 years (body height 173.4 ± 3.7, body mass 74.9 ± 8.6 kg, body fat 12.1 ± 1.9, training experience 5.7 ± 1.3 years) volunteered to participate in this study. They performed a maximal graded aerobic test on a motor-driven treadmill to determine maximum oxygen uptake (VO_2max_), oxygen uptake (VO_2AT_) and heart rate (HR_AT_) at the anaerobic threshold, and maximal heart rate (HR_max_). Additionally, VO_2_ and peak HR (HR_peak_) were recorded following each boxing round. Results showed no significant differences between VO_2max_ values derived from the treadmill running test and VO_2_ outcomes of the simulated boxing contest (*p* > 0.05, *d* = 0.02 to 0.39). However, HR_max_ and HR_peak_ recorded from the treadmill running test and the simulated amateur boxing contest, respectively, displayed significant differences regardless of the boxing round (*p* < 0.01, *d* = 1.60 to 3.00). In terms of VO_2_ outcomes during the simulated contest, no significant between-round differences were observed (*p* = 0.19, *d* = 0.17 to 0.73). Irrespective of the boxing round, the recorded VO_2_ was >90% of the VO_2max_. Likewise, HR_peak_ observed across the three boxing rounds were ≥90% of the HR_max_. In summary, the simulated 3 × 3-min amateur boxing contest is highly demanding from a physiological standpoint. Thus, coaches are advised to systematically monitor internal training load for instance through rating of perceived exertion to optimize training-related adaptations and to prevent boxers from overreaching and/or overtraining.

## 1. Introduction

Amateur boxing is one of the oldest combat sports and it has often been referred to as a “noble art” sport [1]. Boxing is characterized by a stand up fist fight between two opponents of equal weight category [2]. According to the amateur international boxing association [2], an amateur boxing match consists of three rounds, 3-min each with a 1-min rest in-between. Thus, amateur boxing is characterized by intermittent bouts of high-intensity activities interspersed with moments of active and passive recovery [3]. During simulated amateur boxing contests, Davis et al. [4] demonstrated an activity-to-rest ratio of 9:2. This ratio emphasizes the high physical and physiological demands of amateur boxing. In this regard, a narrative review [1] reported that a well-developed aerobic fitness level represents an important performance determinant in amateur boxing to cope with the high metabolic demands of the combat and to allow recovery within and between rounds and/or contests. Of note, the anaerobic metabolism provides energy for short high-intensity bursts during the contest which could have match decisive character [5]. Moreover, it has previously been reported that high levels of muscle strength and power are key components of sporting success in amateur boxing [1].

To appropriately train boxers, knowledge on the dominating metabolic demands during boxing is needed. Such knowledge may help coaches and strength and conditioning professionals to effectively design specifically tailored training interventions that are in accordance with the demands of the sport. Even though a number of studies have already examined the physiological demands of amateur boxing, these studies suffer from methodological limitations (e.g., different amateur boxing exercises [punching bag, pad-work, simulated contest], participants’ training background) and findings were controversial [5]. For instance, with the participation of international level male amateur boxers aged 21 ± 2 years it has been shown that HR values range from 180 to 200 beats·min^−1^ during a sparring activity [6]. During pad-work (i.e., punching on a partner’s pads or mitts) and with the participation of male novice amateur boxers aged 24 ± 4 years^.^, HR values ranged from 184 to199 beats·min^−1^ [3]. However, for punching bag drills, a wider HR range (145 to 195 beats·min^−1^) has been reported [5,7,8]. In terms of blood lactate concentration [La], values between 9 to 12 mmol·L^−1^ have been observed following amateur boxing contests in national level male boxers aged 21 ± 3 years [9]. For pad-work, [La] amounted to 4 mmol·L^−1^ in experienced male amateur boxers [5].

In terms of oxygen uptake, it is difficult to measure VO_2_ during an amateur boxing contest. This is due to the nature of the boxing activity (i.e., punches to the face) which makes it impossible to record gas kinetics. Thus, in previous research, VO_2_ was tested either during a simulated boxing contest [4,5], pad-work [10], or punching bag drills [5]. For instance, Davis et al. [4] examined VO_2_ in a sample of novice boxers during a 3 × 2-min simulated amateur boxing contest using a breath-by-breath gas analyzer and observed that the metabolic profile of amateur boxing is mainly aerobic. With respect to pad-work, Morita et al. [11] reported a mean VO_2max_ of 48.2 ± 3.8 mL·min^−1^·kg^−1^ in six college boxers aged 19 years. Additionally, Finlay et al. [12] showed VO_2_ values of 43.8, 43.3, and 43.5 mL·min^−1^·kg^−1^for the 1st, 2nd, and 3rd round, respectively, using a breath-by-breath portable metabolic analyzer in male elite amateur boxers aged 21 ± 2 years. Concerning punching bag drills, Morita et al. [11] reported VO_2peak_ values of 52.5 ± 7.1 mL·min^−1^·kg^−1^using Douglas bags in male amateur boxers aged 19 ± 1 years. In view of the different methodological approaches and the heterogeneous outcomes, it is imperative to re-assess VO_2_ costs during amateur boxing. More specifically, only a few studies exist that have examined the metabolic demands of the 3 × 3-min bout format in elite amateur boxers [12,13]. The only study that looked at the energy cost of boxing [4] was conducted in a 3 × 2-min simulated bout format with novice male boxers. However, the 3 × 2-min boxing contest format is no longer practiced by AIBA on the elite level [2]. Therefore, the aim of this exploratory study was to examine physiological responses to a semi-contact 3 × 3-min format amateur boxing combat simulation in elite level male boxers. Knowledge from this study can be used to optimize training prescription of elite amateur boxers.

## 2. Materials and Methods

### 2.1. Participants

A total of 11 elite-level male amateur boxers (mean ± SD; age 21.4 ± 2.1 years, body height: 173.4 ± 3.7 cm, body mass: 74.9 ± 8.6 kg, body fat: 13.1 ± 1.6%, training experience: 5.7 ± 1.3 years) from different weight categories voluntarily participated in the study. They were regularly engaged in national competitions during the last 2 years. The mean number of boxing contests performed at the national level by the recruited boxers ranged from 12 to 27. Specifically, the boxer’s sample included three light-welterweight (64 kg), one welterweight (69 kg), three middleweight (75 kg), two light-heavyweight (81 kg), one heavyweight (91 kg), and one super-heavyweight (+91 kg). They were involved in the same training programme which was planned and supervised by the same coach. None of them were involved in any weight loss procedures during the experimental period. Before starting the measurements, all participants responded to a health history and physical activity willingness questionnaire. The questionnaire inquired into any medical issues that might prevent the participant from being included in the study. After being fully informed about the benefits and possible risks of the experimental procedures, all participants signed an informed written consent before starting the study. This study protocol was approved by the local ethical review board for use of human participants of the respective department and was in accordance with latest Declaration of Helsinki.

### 2.2. Experimental Design

This is a cross-sectional study that aimed to examine physiological responses to a semi-contact 3 × 3-min format amateur boxing combat simulation in elite level male boxers. The outcomes of this study may help practitioners to figure out the intensity of 3 × 3-min format amateur boxing combat activity using valid physiological parameters (i.e., VO_2_ and HR). Consequently, this may constitute a useful guideline for coaches to design training intervention that mimics the physiological strain experienced during the match. This may contribute to optimizing performance gains. Participants were instructed to avoid any kind of strenuous activities 48 h before the measurements. They were also instructed to get light meals 2 to 3 hours before measurements and to avoid caffeine ingestion 24 h before and during the measurement days. All testing sessions were conducted between 9:00 a.m. and 11:00 a.m. under similar environmental conditions (temperature 17–24 °C, humidity 41–59%, barometric pressure 757–759 mmHg). Experimental conditions included treadmill running and a 3 × 3-min simulated amateur boxing contest on two different occasions with a minimum of 3 and a maximum of 7 days in-between.

### 2.3. Treadmill Running Test

A maximal graded aerobic running exercise was administrated on a treadmill. After 15-min warm-up, participant started an incremental running test using a motor-driven treadmill (Parker brand, LET Medical Systems Corp, Miami Lakes, FL, USA) to assess maximal oxygen consumption (VO_2max_), maximal HR (HR_max_), and oxygen uptake (VO_2AT_) and heart rate (HR_AT_) at the anaerobic threshold. Running intensity progressively increased by 2 km·h^−1^ every 3 min, starting from 6 km·h^−1^ till volitional exhaustion. The treadmill slope was set at 1% to compensate for the lack of air friction [14]. A verbal encouragement was given to each participant to reach maximal effort during the test. Attainment of VO_2max_ was considered when respiratory exchange ratio (RER) was over 1.10 and HR ± 10 beats·min^−1^ of predicted maximum HR (220 − age) [15]. Heart rate was recorded at 10 s intervals using a pulse monitor (Polar Sport-tester TM PE 3000; Polar Electro, Kempele, Finland) [16,17]. Expired gases were sampled and examined by a telemetric respiratory gas analysis system (K4b2, Cosmed, Rome, Italy). Before starting the measurement, the gas-analysis system was calibrated with ambient air (O_2_: 20.93% and CO_2_: 0.03%) and a gas mixture (O_2_: 16.00% and CO_2_: 5.00%) and the turbine was calibrated with a 3-L syringe (Hans Rudolph Inc., Dallas, TX, USA) according to the manufacturer’s instructions. The average of VO_2_ acquired during the last 30 s before volitional exhaustion was considered as the VO_2max_ of the boxer. The VO_2AT_ was calculated using the gas exchange V-slope in addition to the ventilatory techniques, as described previously [18,19].

### 2.4. 3 × 3-min Simulated Amateur Boxing Contest

The simulated boxing contests were carried out on an accredited standard boxing ring following a 15-min warm-up (10 min running, 2 min of full-body stretching, and 3 min of shadow boxing). During the simulated boxing contest, oxygen consumption (VO_2_) and peak HR (HR_peak_) were recorded. Of note, VO_2_ was measured throughout the simulated boxing contest using a portable breath-by-breath gas analyzer (K4b2, Cosmed, Rome, Italy), whereas HR_peak_ was immediately recorded after the end of each round.

As in an official boxing contest, all participants wore their bandages (4.5 m length, 5 cm width; Adidas, Berlin, Germany) and their boxing gloves (10 oz; Adidas, Berlin, Germany). Contest simulation consists of a series of offensive and defensive skills against handheld pads (10 inches; Adidas, Berlin, Germany). The simulated boxing contest was designed from a previously analyzed video footage of amateur boxing 3 × 3-min format bouts [20] (Table 1). Pads were held by a qualified amateur boxing association coach. The same coach was used for all boxing contests. Even though participants were not supposed to react to real attack actions, they were compelled to perform defensive movements against the coach throwing punches using the pads. The whole contest simulation lasted for 15 min: 2 min pre-contest resting period and then three boxing rounds with 1 min recovery time in-between and a final 2 min of rest post-contest.

### 2.5. Statistical Analysis

Data are presented as mean values and standard deviations. Data were tested for normal distribution using the Shapiro-Wilk’s test. To compare physiological variables recorded during the three boxing rounds, one-way ANOVA with repeated measures on boxing rounds was used. Post-hoc analyses were conducted using Bonferroni correction. Paired sample *t*-tests were used to explore the differences between physiological variables recorded during the treadmill running test and the simulated amateur boxing context. To determine the magnitude of the difference, effect sizes were determined by calculating Cohen’s *d* values [21]. The same author classified *d* values as small (*d* < 0.50), medium (0.50 ≤ *d* < 0.80), and large effects (*d* ≥ 0.8). The level of significance was set at *p* ≤ 0.05. All statistical analyses were performed using SPSS 20.0 (SPSS, Inc., Chicago, IL, USA). 

## 3. Results

The mean values relative to VO_2max_, VO2_AT_, HR_max_, and HR_AT_ recorded during the treadmill running test are presented in Table 2. Irrespective of the boxing round, paired sample t test showed no significant differences between VO_2max_ values retained from the treadmill running test and VO_2_ outcomes of the simulated boxing contest (*t* = −2.00 to 0.06, df = 10, *p* > 0.05, *d* = 0.02 to 0.60). However, HR values recorded during the treadmill running test and the simulated boxing contest displayed significant differences regardless of the boxing round (*t* = −33.10 to −9.69, df = 10, *p* < 0.01, *d* = 3.00 to 11.00) being higher following the treadmill running test. 

The physiological outcomes in terms of VO_2_ (mL·min^−1^·kg^−1^), %VO_2max_, HR_peak_ (beat·min^−1^), and %HR_max_ recorded during the simulated amateur boxing contest are shown in Table 3. With respect to VO_2_ outcomes, no significant between-rounds differences were observed (F_(2-32)_ = 1.71, *p* = 0.19, *d* = 0.17 to 0.73). Irrespective of the boxing round, the recorded VO_2_ represented >90% of the VO_2max_ (Table 3). In terms of HR_peak_, significant between-rounds differences were reported (F_(2-32)_ = 27.50, *p* < 0.001, *d* = 1.07 to 1.58). Higher HR_peak_ values were recorded during round 3 compared to round 2 and 1 (both *p* < 0.01). Similarly, higher HR_peak_ outcomes were observed during round 2 compared to round 1 (*p* < 0.01). HR_peak_ values observed across the three boxing rounds were ≥90% of the HR_max_ (Table 3). 

## 4. Discussion

The aim of this study was to examine the physiological responses to a semi-contact 3 × 3-min format amateur boxing combat simulation in elite level male boxers. The main findings of this study demonstrated that, although under simulated conditions, 3 × 3-min amateur semi-contact boxing contest induced high-intensity physiological strain approaching the maximal level. Specifically, the designed simulated competitive contest-elicited high cardio-respiratory responses (e.g., VO_2_ and HR), suggesting marked involvement of the oxidative metabolism during simulated amateur boxing contests.

Heart rate is widely accepted as an accurate indicator of exercise intensity [22]. Generally, athletes with better “aerobic” physical conditions have higher resting bradycardia [23] and smaller rise in HR at any level of effort when compared to individuals at lower competitive levels [24]. The present findings showed high cardiovascular responses during amateur boxing contest simulation. In fact, HR_peak_ recorded for the 1st, 2nd, and 3rd round were 90%, 91%, and 92% of HR_max_, respectively. This is indicative of the high physiological stress of amateur boxing activity. Previous studies conducted on simulated amateur boxing contests reported lower HR responses across the three boxing rounds (162–174 beat·min^−1^) [4,12]. The higher HR values observed in the current study are likely to be due to the longer rounds duration compared, for instance, to the study of Davis et al. [4] (3 min vs. 2 min, respectively). It is noteworthy that HR_peak_ showed a progressive increase throughout the whole amateur simulated boxing contest, with higher values recorded during the 2nd and the 3rd rounds in comparison with the 1st round [4,12]. This phenomenon was not evident for the other gathered physiological variables (i.e., VO_2_). This observation suggests that there was a kind of dissociation between HR and the metabolic requirements of the amateur simulated boxing contest being performed, which caused an increase in HR over the real metabolic engagement [25]. During the amateur simulated boxing contest, the HR_peak_ illustrated an unexpected higher rate of changes, especially in comparison with the ones of the VO_2_. The reason behind this may partly be related to the excessive CO_2_ production due to the high-intensity effort throughout the consecutive rounds. Moreover, the 1-min between-round intervals are not adequate to achieve sufficient recovery. Indeed, Anderson et al. [26] have shown that to sufficiently recover from an exhaustive activity, athletes may require between 48 h to 72 h. 

Amateur boxers’ cardiorespiratory fitness is one of the most relevant aspects of competitive performance [4,6,27]. In this context, well-developed aerobic fitness might help the boxer to maintain repetitive high-intensity actions within a boxing contest, also to accelerate the recovery process and to keep the boxer fit until the end of the contest [1,28]. The overall VO_2max_ outcomes reported in the scientific literature for male boxers vary between 49 and 65 mL·min^−1^·kg^−1^ [1]. Participants’ mean VO_2max_ values recorded from the treadmill running test (55.45 mL·min^−1^·kg^−1^) fall within the range of values reported in the literature. Results of the current study showed that VO_2_ outcomes recorded across the three simulated boxing rounds were >90% VO_2max_. More specifically, the recorded VO_2_ values were either very close or exceeded the maximum level derived from the maximal graded aerobic test. This particular observation emphasizes the high physiological stress of amateur boxing activity.

The simulated amateur boxing contest undertaken in the present study showed VO_2_ values of 55.3, 53.8, and 50.4 mL·min^−1^·kg^−1^ for the 1st and 2nd, and 3rd round, respectively. These VO_2_ outcomes are higher than the outcomes recorded during punching routine activities in the previous study [12] where values of 44, 43, and 44 mL·min^−1^·kg^−1^ were recorded for the 1st and 2nd, and 3rd round, respectively. It is noteworthy that more detailed physiological analyses were not performed because of the rounds’ short duration (i.e., 3 min).

There are some limitations that have to be acknowledged. First, there was a relatively small number of recruited participants, although a small sample size could be preferable to increase sample’s homogeneity; however, we were unable to get more participants of such a competitive level. Second, is the simulated character of the amateur boxing fights. In this context, given the nature of boxing activity and rules’ constraint, using a portable breath-by-breath gas analyzer during an official boxing contest was quite impracticable. 

## 5. Conclusions

This is the first study to analyze the physiological responses of 3 × 3-min semi-contact amateur boxing combat in elite-level male boxers. Taken together, findings of the present study indicated that the semi-contact 3 × 3-min amateur boxing format is highly stressful from a metabolic standpoint for elite-level boxers. Therefore, coaches and strength and conditioning professionals are advised to systematically monitor internal training load for instance through rating of perceived exertion to optimize training-related adaptations and to prevent boxers from overreaching and/or overtraining. Given that the scientific literature on female amateur boxing is scarce, future research is needed to better characterize females’ activity from a physiological point of view.

## Figures and Tables

**Table 1:** Total punches and boxing combinations used in the simulation.

Variable	Round 1	Round 2	Round 3
Total Punches	54.4	51.2	52.3
Lead Hand Punch	28.5	26.6	24.7
Rear Hand Punch	22.8	20.8	21.6
Total Boxing Combinations	27.7	23.3	22.3
2 Punches Combinations	13.4	11.9	11.5
3 or more punch combinations	5.2	4.8	3.9
Defense with arm	5.1	4.2	4.1
Defense with foot	3.7	2.6	2.2
Defense with trunk	2.8	1.7	2.2

**Table 2:** Physiological responses during the treadmill running test (*N* = 11).

Physiological Variables	VO_2max_ (mL·min^−1^·kg^−1^)	VO_2max_ (mL·min^−1^)	VO2_AT_ (mL·min^−1^·kg^−1^)	VO2_AT_ (mL·min^−1^)	HR_max_ (beat·min^−1^)	HR_AT_ (beat·min^−1^)
Mean	55.45	4113.22	39.03	2526.68	196.27	179.81
SD	6.86	359.27	2.93	240.60	1.68	2.83

VO_2max_ = maximum oxygen uptake; VO2_AT_ = oxygen uptake at anaerobic threshold; HR_max_ = maximum heart rate; HR_A_T = heart rate at anaerobic threshold.

**Table 3:** Physiological responses to the simulated amateur boxing contest (N = 11).

Physiological Variables	Round 1 (Mean ± SD)	Round 2 (Mean ± SD)	Round 3 (Mean ± SD)
VO_2_ (mL·min^−1^·kg^−1^)	55.3 ± 4.4	53.8 ± 6.5	50.4 ± 7.9
VO_2_ (%max)	101 ± 0.1	99 ± 19.4	92 ± 15.1
HR_peak_ (beat·min^−1^)	176 ± 4.1	179 ± 1 ^§^	182 ± 5 ^¥,¶^
HR (%max)	90 ± 2.2	91 ± 1.4	92 ± 2.3

VO_2_ = oxygen uptake; VO_2_ (%max) = percent of maximum oxygen consumption; HR_peak_ = peak heart rate; HR (%max) = percent of maximum heart rate; ^§^ = significantly different from round 1; ^¥^ = significantly different from round 1; ^¶^ = significantly different from round 2.
